# The characteristics of ctDNA reveal the high complexity in matching the corresponding tumor tissues

**DOI:** 10.1186/s12885-018-4199-7

**Published:** 2018-03-23

**Authors:** Nong Yang, Yi Li, Zhidong Liu, Hao Qin, Duanming Du, Xinkai Cao, Xiaoqing Cao, Jun Li, Dongge Li, Bo Jiang, Lincan Duan, Haiyan Yang, Zhenghua Zhang, Hao Lin, Jianying Li, Zhenhua Yang, Lei Xiong, Hua Shen, Lizhu Lin, Fugen Li

**Affiliations:** 1 Lung Cancer and Gastrointestinal Unit, Department of Medical Oncology, Hunan Cancer Hospital, Changsha, China; 2 Department of Oncology, Yunnan Province Traditional Chinese Medicine Hospital, Kunming, China; 3 Second Department of Thoracic Surgery, Beijing Chest Hospital, Capital Medical University, Beijing, China; 4 The Research and Development Institute of Precision Medicine, 3D Medicine Inc, Shanghai, China; 5 Department of Interventional Radiology, Shenzhen Second People’s Hospital (First Hospital of Shenzhen University), Shenzhen, China; 6 Department of Cadre’s Medical Oncology, The Third Affiliated Hospital of Kunming Medical University(Yunnan Cancer Hospital), Kunming, China; 7 Department of Thoracic Surgery, The Third Affiliated Hospital of Kunming Medical University, Yunnan Cancer Hospital, Kunming, China; 8 Department of oncology, Jing’An District Centre Hospital of Shanghai (Huashan Hospital Fudan University Jing’An Branch), Shanghai, China; 9 Department of Oncology, Huashan Hospital north, Fudan University, Shanghai, China; 10 Department of Oncology, Nantong Tumor Hospital, Nantong, China; 11 Department of Respiratory Medicine, Nanjing First Hospital, Nanjing Medical University, Nanjing, China; 12 Department of Oncology, Sir Run Run Hospital, Nanjing Medical University, Nanjing, China; 13 Department of Oncology, First Affiliated Hospital of Guangzhou University of Traditional Chinese Medicine, Guangzhou, China

**Keywords:** Next-generation sequencing, Precision medicine, Capture-base sequencing, ctDNA, Lung cancer, UC-seq, Short ctDNA, Blood tumor mutational burden

## Abstract

**Background:**

Next-generation sequencing (NGS) is an efficient and sensitive method to detect mutations from ctDNA. Many features and clinical conditions could significantly affect the concordance between ctDNA and corresponding tumor tissues. Our goal was to systematically investigate the critical factors contributing to different concordance between ctDNA and corresponding tumor tissues.

**Methods:**

We recruited two groups of IIIB or IV lung cancer patients: The standard group to evaluate the accuracy of our method and the concordance between ctDNA and tumor tissues, and the study group with various clinical conditions. We applied our unique identification (UID) indexed capturing-based sequencing (UC-Seq) to ctDNA samples, and confirm the results by Droplet digital PCR (ddPCR).

**Results:**

Considering mutations detected from NGS of tumor tissues as golden standard, UC-Seq achieved overall 93.6% sensitivity for SNVs and Indels, and 0.8 Pearson correlation between tumor TMB and bTMB. Efficacious treatments, long sampling date (more than 2 weeks) between tumor tissues and ctDNA and low concentrations of cfDNA (less than 9 ng/ml) could significantly decrease the concordance between ctDNA and tumor tissues. About 84% mutations showed shorter mutant fragment length than that of wild-type fragments, and the AFs of mutations could be significantly enriched in small-size ctDNA.

**Conclusions:**

In late-stage lung cancer patients, ctDNA generally has high concordance with tumor tissues. However it could be significantly affected by three clinical conditions which could dynamically change the content of ctDNA. Moreover, the detection limit could be further extended by enriching small-size ctDNA in the preparation of samples.

**Electronic supplementary material:**

The online version of this article (10.1186/s12885-018-4199-7) contains supplementary material, which is available to authorized users.

## Background

Among all cancers, lung cancer has the highest incidence and mortality per year, which becomes a worldwide problem of public health [1–3]. Though patients with early-stage lung cancer have high overall survival after surgery or stereotactic body radiation therapy, whose 5-year survival can be over 50%, advanced lung cancer patients might not get sufficient benefits from similar treatments [4]. Lung cancer has been proved to be a highly heterogeneous disease, and over 85% lung cancer patients are diagnosed as non-small cell lung cancer (NSCLC) [5]. It is estimated that about 69% of advanced NSCLC patients possess at least one potential actionable drug target, which enabled targeted therapies [4]. Hence, based on personal tumor profiles of DNA aberrations, the concept of precision medicine arises to individually strategize treatments in advanced cancers, which has been widely recognized.

Targeted drugs usually target one or several DNA aberrations, which requires appropriate biopsies and technology to identify relevant biomarkers, including single nucleotide variations (SNVs), insertion and deletions (Indels), gene fusions, copy number variations (CNVs) and abnormal expressions [4]. The detection method could be categorized to two classes: 1. PCR-based techniques, which could detect single DNA aberration per reaction at extremely high sensitivity, including Amplification-refractory mutation system (ARMS), droplet digital PCR (ddPCR) and BEAMing; 2. Sequencing-based techniques, which could detect multiple aberrations simultaneously, including whole genome sequencing (WGS), amplicon sequencing and target capture sequencing.

Though tissue biopsy is a well-accepted practice in targeted therapies, circulating tumor DNAs (ctDNAs), which are released from dead tumor cells to the blood stream, have attractive advantages over tissue biopsy in the applications of precision medicine, such as the sampling convenience and dynamic monitoring. However, the proportion of ctDNA in blood is extremely low, which requires super sensitive methods to detect mutations of allelic frequencies as low as 0.1% [6]. The performance of ctDNA detection of lung cancer patients varies according to methods and tumor stages. ctDNAs from late-stage lung cancer generally have higher sensitivity (from 74% to 85%) [7–9] to detect tissue-matched mutations than that from early stages (53.8%) [10] by targeted next generation sequencing (NGS) in the past 2 years. Recently, *AM Newman* proposed a digital error suppression process with barcoding technique to further increase the sensitivity of mutation detection from ctDNA to 93% [11]. Based on this concept, we conducted a multiple center study on 131 tumor-ctDNA pairs of samples from late-stage (IIIB and IV) lung cancer patients to evaluate the utility of ctDNA targeted NGS in precision medicine. We systematically investigated the accuracy and the specificity of mutation detection from ctDNA and identified several key factors that might significantly affect the results. Furthermore, it was reported that ctDNA molecules from tumor cells were shorter than the cell-free molecules from normal cells in a small sample set [12]. Thus we extended the analysis on the length of ctDNA fragments and its association with clinical features.

## Methods

### Patient selection and sample collection

Several criteria were applied to the standard group of the patients included in this study: 1. the patients were diagnosed with lung cancers at the stages of IIIB or IV; 2. the patients were treatment naive; 3. the blood samples were collected before or after acquiring tumor tissues within 14 days; 4. the tumor tissue samples were collected by either percutaneous needle biopsy or surgery, but for the surgery patients, the blood samples were collected at least 1 day before surgery. For each patient, 8-10 ml blood was drawn by venipuncture and was stored in Cell-Free DNA™ BCT (BCT) (Streck Inc., Omaha, NE). The paired tumor tissues were fixed in formalin. The samples were shipped to the Research Center of 3DMed under a constant room temperature. The time between sample collection and processing was less than 48 h.

### DNA extraction

To separate plasma, the blood in STRECK tubes was centrifuged at 1600 g for 20 min at room temperature. The blood was separated into three layers: the upper layer was plasma, the middle buffy coat was white cells, and the lower layer was red blood cells. Afterwards the plasma layer was carefully transferred to a new 1.5 ml Eppendorf tubes, followed by a room-temperature centrifuge at 16000 g for 10 min to remove the residual cells and debris. The buffy coat was then transferred to a new tube for genomic DNA (gDNA) extraction.

The tumor tissues were firstly subjected to H & E staining to determine the percentage of tumor cells. The tumor cell percentage should be over 20% to be considered as a qualified sample [13]. The gDNAs of FFPE tumor tissues and white blood cells were extracted by the DNeasy Tissue or Blood Kit (Qiagen) respectively following the standard protocols. Cell-free circulating DNAs in plasma were extracted by QIAamp Circulating Nucleic Acid Kit (Qiagen) following the standard protocols. The DNA concentrations were determined by Qubit dsDNA HS Assay Kit (Life Technologies). Genomic DNAs were fragmented to a size ranging from 200 bp to 400 bp using the Covaris S2 Sonolab (Covaris).

### Library preparation, target capture and DNA sequencing

gDNA libraries were established by KAPA Hyper Prep Kit (KAPA Biosystems) according to the manual. The cfDNA libraries were prepared by Accel-NGS 2S Plus DNA Library Kit (SWIFT) with unique identifiers (UIDs, also called barcoding technology) to tag individual DNA molecules. The concentrations of libraries were determined by Qubit, and the size distributions of libraries were analyzed by Caliper.

One to four libraries with different sample indexes were firstly pooled together, where the total DNA amount was 1 μg. The pooled DNAs were mixed with 2 ul of DNA blocker (Integrated DNA Technologies) and 5 ul of human Cot-1 DNA (Invitrogen), and then dried by a vacuum concentrator (Themofisher). The dried mixture was dissolved in a 15 ul hybridization buffer supplied by the hybridization of xGen Lockdown Probes kit (Integrated DNA Technologies), and thereafter the targeted DNAs were captured following the standard protocol by a customized set of biotinylated DNA probes. The captured DNAs were then amplified by PCR, whose final DNA concentrations were determined by Qubit and the DNA sizes were analyzed by Caliper.

1.6–1.7 Pmol/L captured libraries were loaded into the NextSeq500 (Illumina) to run 75 bp paired-end sequencing with Illumina version 4 sequencing kits according to the manufacturer’s instructions.

### Bioinformatic analysis and statistics

The paired-end reads were mapped by BWA [14] MEM algorithm. SNVs were called by MuTect [15] with default parameters. Small insertions and deletions were called from the union of Varscan 2 [16] and Pindel [17] with default parameters. Fusions were called by self-developed scripts with at least 5 pairs of reads spanned over the breakpoints between two partner genes. The CNVs of tumor tissues were calculated by BIC-seq2 [18] with default parameters, and the CNVs of ctDNA samples were called by a method reported by Jacob J. Chabon et al. [19]. All mutations were manually reviewed using IGV [20] to further eliminate false-positive results. The probability density distributions of mutant and wild-type fragments were calculated by Gaussian kernel smoothing using StatsModels 0.8.0.

### ctDNA library size fractionation

To separate the smaller and larger DNA fragments in library by electrophoresis, the library DNA was run in 2% agarose gel. The DNA fractions with the sizes of 200-300 bp and 350-600 bp were sliced and stored in different tubes, followed by a purification of Qiaquick gel extraction kit (Qiagen). The DNA concentrations were determined by Qubit dsDNA HS Assay Kit (Life Technologies).

### Droplet digital PCR

The droplet digital PCR was performed on libraries by the droplet digital PrimePCR™ (BioRad) on the BioRad QX200 droplet digital PCR system.

## Results

### The concordance of mutations between ctDNA and its corresponding tumor was high

To examine how the performance of the unique identification (UID) indexed capturing-based sequencing (UC-Seq) method was, a total of 56 pairs of tumor-ctDNA samples as the standard group were collected from a few of major hospitals across China. The clinical features of patients were summarized in Table 1. Only samples of stage IIIB (28.6%) or IV (71.4%) patients classified by American Join Committee on Cancer (AJCC) criteria were collected. The tumor tissue samples were gathered from either primary sites (67.9%) or metastatic sites (32.1%) depending on the availability. The samples comprised lung adenocarcinoma (69.6%), lung squamous cell carcinoma (16.1%), and other minor types of lung cancers (14.3%). All patients were treatment naive, whose blood samples were collected at least 1 day before surgery or biopsy, or within 14 days after tissue biopsy.

**Table 1:** Clinical features of patients in the standard group

	Mean	SD
Age (Years)	40.4	12.1
Sampling interval (Days)	2.6	4.0
	Number of samples	Proportions
Sex		
Male	30	53.6%
Female	26	46.4%
Cancer type		
LUAD	39	69.6%
LUSC	9	16.1%
Others	6	10.7%
Unknown	2	3.6%
Tumor site		
Primary	38	67.9%
Metastatic	18	32.1%
AJCC stage		
IIIB	16	28.6%
IV	40	71.4%

**Fig. 1:**
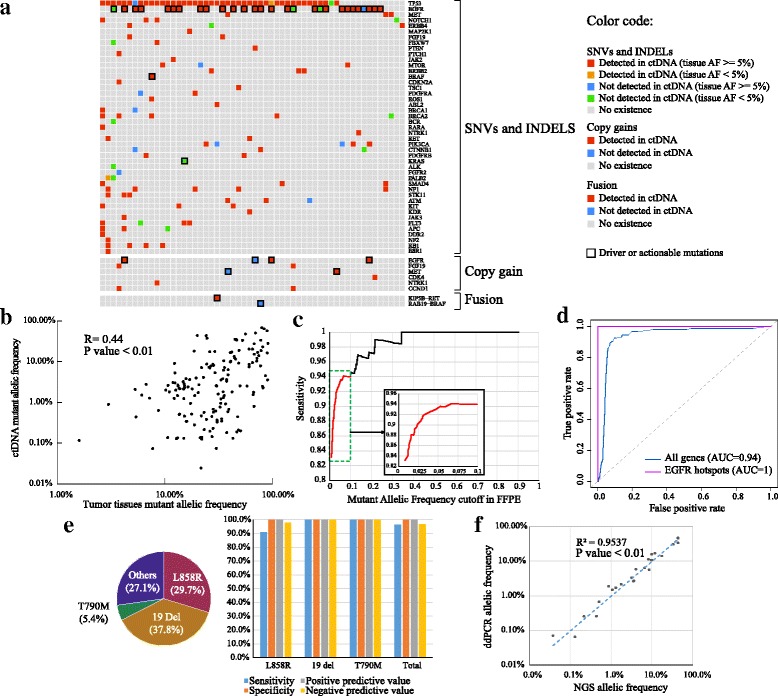
ctDNA had high concordance to corresponding tumor tissues from late-stage lung cancer patients. **a** The summary of mutations in the standard group of patients. **b** The Pearson correlation of allele frequencies (AFs) between ctDNA and tumor tissues. **c** The curve of ctDNA sensitivity to detect tumor-matched SNVs and Indels at different cutoffs of tumor allelic frequency. **d** The receiver operating characteristic (ROC) curve to detect tumor-matched SNVs and Indels from ctDNA. **e** The sensitivity of ctDNA in detecting EGFR hotspot mutations. **f** The correlation of AFs between ctDNA and ddPCR

Both ctDNA and tumor tissues were sequenced by UC-Seq of a customized panel comprising 63 full-length targeted-therapy related genes, which could simultaneously detect single nucleotide variations (SNVs), small insertions and deletions (Indels), copy number variations (CNVs) and gene fusions. The ctDNA samples were sequenced at the coverage of 10,000× with barcodes, and tumor tissue samples were sequenced at the coverage of 800× without barcodes. After deduplication of reads, the average coverage was 3000× for ctDNA and 500× for tumor tissues respectively. The minimum coverage was 2000× for ctDNA and 300× for tumor tissues. If the coverage of samples was below the minimum coverage, the corresponding libraries would be re-sequenced and the reads would be pooled until the final coverage was above the minimum coverage. A total of 145 single nucleotide variations (SNVs), 34 short insertions/deletions (Indels), 2 fusions and 12 copy number gains whose gain ratios were larger than 3.5, were identified in tumor tissue samples. The sensitivity of detecting those SNVs and Indels from tumor tissues in ctDNA significantly increases from 63.5% to 83.2% (*p* value < 0.01) (Additional file 1: Figure S1A) from non-barcoding technique to barcoding technique. The barcoding technique detected about 30% more tissue-matched mutations, especially those whose allelic frequencies (AF) in ctDNA were below 0.5% (Additional file 1: Figure S1B). Among those excessive mutations detected by barcoding technique, 25.7% of the mutations were actionable. The overall matching result between blood samples and tumor tissue samples were shown in Fig. 1a.

In this study, we considered the detection of tumor tissues as golden standard. The AFs of SNVs and Indels in ctDNA didn’t have strong correlation with those in tumor tissues (Fig. 1b). The sensitivity of tumor-matched mutation detection in ctDNA was affected by their mutant AFs in the tissues (Fig. 1c). The sensitivity could reach 93.6% when the mutant AFs in tissue were > = 5%, while the sensitivity dropped rapidly to only 9.1% when the mutant AFs in tissue were below 5%. Therefore, we set AFs of mutations in tumor tissues to 5% as a new cutoff for the following analysis in this study. We found that ctDNA from lung squamous cell carcinoma showed slightly higher sensitivity (95.5%) to detect tumor-matched mutations than that from lung adenocarcinoma (91.7%), but it was not statistically significant (*p*-value = 1.0). Mutations detected in ctDNA had slightly higher concordance with the tumor tissues from metastatic sites (98.1%) than those from primary sites (91.3%) without significant *p*-value (0.17). And ctDNA had slightly better sensitivity (93.7%) to detect tumor-matched mutations in IIIB patients than that in IV patients (93.3%) without significant *p*-value (1.00). The receiver operating characteristic (ROC) curve whose area under curve (AUC) was 0.94, indicated an exceptional overall prediction power of tumor-matched mutations detected in ctDNA (Fig. 1d). Besides, the prediction power of EGFR was also remarkable (Fig. 1d, e). The detection of EGFR hotspots (L858R and exon 19 deletion) can reached over 90% sensitivity and 100% specificity. Moreover, the AFs of EGFR hotspots calculated by UC-Seq had 0.95 Pearson correlation (*p* value < 0.01) with those measured by ddPCR (Fig. 1f). From the results above, SNVs and Indels detected from ctDNA by UC-Seq are highly concordant with its corresponding tumor tissues.

The detection of copy number variation (CNV) was influenced by the ratio of copy number variations and the proportions of ctDNA in cfDNA (Additional file 2: Figure S2A). We used the maximum mutant AF of somatic mutations in a sample to approximate the proportions of ctDNAs in cfDNAs, and the result showed that the copy gain events from tumors can be effectively detected at high copy gain ratios or at high proportions of ctDNAs in cfDNAs. For copy gain events whose ratios were larger than 3.5, the sensitivity of detection in ctDNA achieved 83.3% (Additional file 2: Figure S2B).

Two tumor tissues with fusion events were integrated in our validation study. The KIF5B-RET fusion event was also detected in ctDNA, but RAB19-BRAF was missing. Since the introns of BRAF contains large amount of short interspersed nuclear element (SINEs), many regions could not be targeted by specific-probes. The breakpoint of the RAB19-BRAF fusion event could happen in the regions where probes couldn’t efficiently cover. Because there were few cases of fusions in our data, we could not thoroughly analyze the detection power of UC-Seq on ctDNA.

### ctDNA presented good correlation of tumor mutational burden (TMB) with tumor tissue

**Fig. 2:**
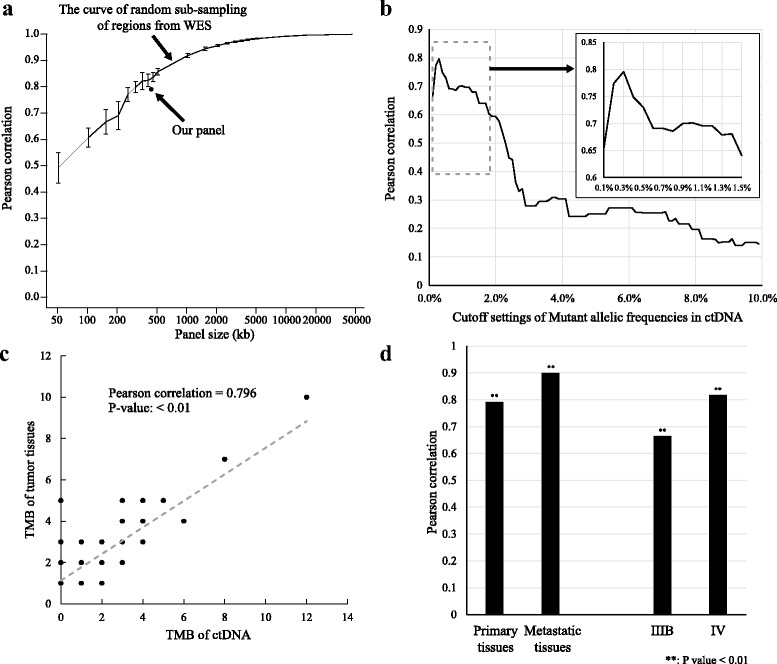
bTMB had high correlation with tumor TMB. **a** The curve of Pearson correlation between different sizes of small panels and WES. **b** The curve of Pearson correlation between bTMB and tumor TMB with different SNV and Indel AF cut-offs in ctDNA. **c** The Pearson correlation between bTMB and tumor TMB with AF cut-off 0.3% in ctDNA. **d** Pearson correlation between bTMB and tumor TMB with different clinical conditions

Tumor mutational burden (TMB) measures the frequency of mutations occurred in all exon regions of protein coding genes. We explored whether the blood TMB (bTMB) from a small panel in ctDNA could properly reveal TMB of the corresponding tumor tissues. Firstly, we evaluated the correlation of TMB between randomly selected small panels of various sizes and whole exome sequencing (WES) with 408 lung cancer tumors from TCGA (Fig. 2a). At each size, we sampled 10 replicates. The Pearson correlation of TMB between a small panel and the WES panel dropped rapidly below 0.9 (*p* value < 0.01) when the panel size was smaller than 1 million base-pairs (bp). The size of our panel for this study was about 490 KB, and the correlation of TMB between our panel and WES was about 0.8 (*p* value < 0.01), which was reasonably good. Thereafter, we compared TMB between tumor tissue and ctDNA. As the data showed in the previous session, the cutoff of mutant AFs in tumor tissues was set to larger or equal to 5%. We took different cutoffs of mutant AFs in ctDNA and plot a curve on their Pearson correlations of TMB with tumor tissues (Fig. 2b). The best correlation was about 0.8 (*p* value < 0.01) and the cutoff of mutant AF in ctDNA was 0.3% (Fig. 2c). The correlation of TMB between tumor tissues and ctDNA was significantly affected by the source of tissues and the tumor stages (Fig. 2d). The correlation was 0.9 (*p* value < 0.01) between tissues from metastatic sites and ctDNA, but only 0.8 between tissues from primary sites and ctDNA. Tissue TMB from IV patients had much better correlation (0.8, *p* value < 0.01) with bTMB from ctDNA than that (0.66, *p* value < 0.01) from IIIB patients. Overall, our data suggested that our panel for bTMB should be sufficient enough to represent the tumor TMB for late stage cancer.

### Various clinical conditions could significantly affect the detection of mutations in ctDNA

Besides the standard group of samples, we also collected 75 samples with various clinical conditions and compared their concordance of SNVs and Indels whose AFs were higher than 5% in tumor tissues between ctDNA and tumor tissues. Firstly, we tried to investigate how treatments would influence the sensitivity of variants detection in ctDNAs. Two types of patients were recruited: 1. Patients who had received surgery and had blood sampled after surgery; 2. Patients who were treated targeted therapies or chemotherapies. Among the samples, 22 patients had blood sampling at least 1 day after surgeries from which tumor tissues were acquired. The ctDNA collected after surgeries still showed high concordance (87%) with the tumor tissues from metastatic sites. However, the concordance with the tumor tissues from primary sites dropped significantly to 41.0% (*p*-value < 0.01). Furthermore, we received five cases who were taking inefficacious chemotherapies or targeted therapies with progress diseases. The ctDNA of the five cases presented high concordance (91.3%) with the tumor tissues. These results indicate that the efficacy of therapies could significantly affect the concordance between ctDNA and tumor tissues. Our result is in line with the current ctDNA NGS detection in clinical application that ctDNA has great potential to monitor the consequences of therapies.

Additionally, we received 13 blood samples that were acquired more than 2 weeks after tumor biopsies from treatment-naive patients, and the patients hadn’t received any therapies before blood sampling. We found a significant difference (*p* value = 0.032) of concordance between IIIB patients and IV patients. Though there were only three IIIB patients in this category, all mutations from tumor tissues could be detected in ctDNA. In contrast, only 54.1% mutations from tumor biopsies of IV patients could be detected in their corresponding ctDNA samples.

### Samples with low cfDNA concentrations had reduced sensitivity to detect mutations

**Fig. 3:**
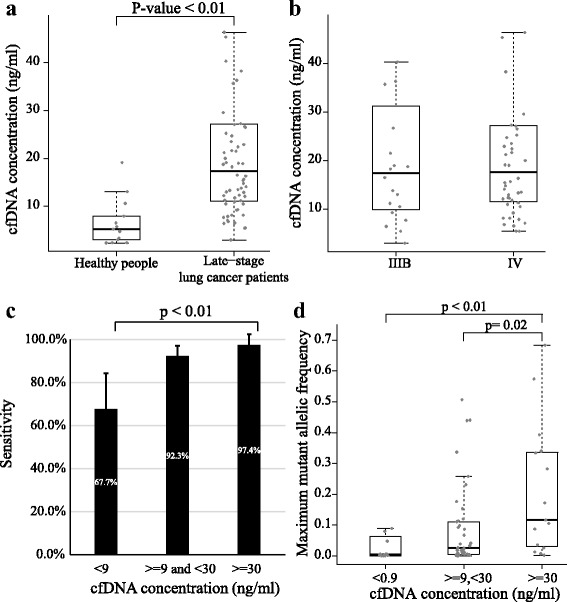
cfDNA concentration could significantly affect the concordance between ctDNA and tumor tissues. **a** The boxplot of cfDNA concentrations between healthy people and late-stage lung cancer patients. **b** The boxplot of cfDNA concentrations between IIIB patients and IV patients. **c** The sensitivity of ctDNA with different cfDNA concentrations. **d** The boxplot of the maximum mutant allelic frequencies in ctDNA samples with different cfDNA concentration

Late-stage lung cancer patients generally had significantly higher cfDNA concentrations than that in healthy people (*p* < 0.01) (Fig. 3a). However, some patients might have low cfDNA concentrations close to the level of healthy people. The IIIB and IV patients had similar distributions of cfDNA concentrations (Fig. 3b). However, considering mutations detected in the paired tumor tissues as golden standard, ctDNA samples from the patients with high cfDNA concentrations showed better sensitivity to detect tumor-matched mutations than those from the patients with low cfDNA concentrations (*p*-value < 0.01) (Fig. 3c). Besides, the AFs of tumor-matched mutations in cfDNA samples with high concentrations (> = 30 ng/ml) were significantly higher than those in cfDNA samples with low concentrations (< 9 ng/ml) (*p* < 0.01) (Fig. 3d). These results suggested that cfDNA concentrations might reveal the death rate of tumor cells or the tumor burden. The samples with low cfDNA concentrations might contain small proportion of ctDNA, and mutation-detection needs technology improvement for the patients whose cfDNA concentrations were lower than 9 ng/ml.

### Most mutant fragments were overall shortened compared with wild-type fragments

**Fig. 4:**
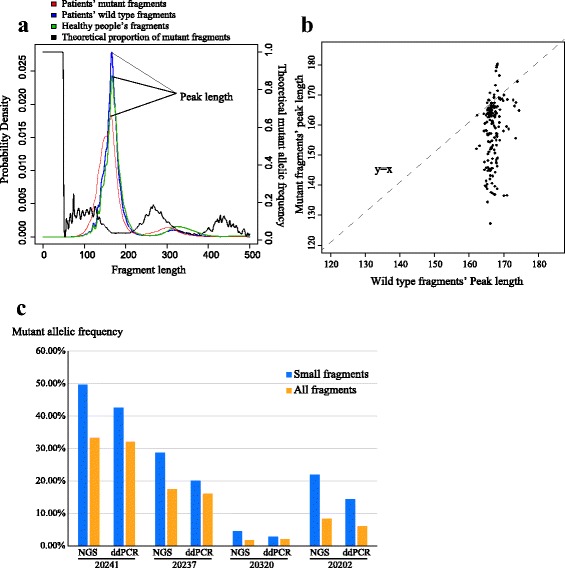
Mutant fragments of most mutations were enriched in small-size ctDNA. **a** The curve of probability density distribution of fragment sizes calculated by Gaussian smoothing. **b** The scatter plot of peak length of all SNVs and Indels with mutant fragment number larger than 20. **c** ddPCR validation of mutant enrichment of EGFR L858R mutation in small-size ctDNA

We further modeled the distributions of fragment lengths by Gaussian smoothing with all 131 samples from patients and 13 healthy participants (Fig. 4a). The curves of wild type fragments, mutant fragments and fragments from healthy participants all showed conventional double-peak pattern. However, the mutant fragments had higher proportions of small fragments (< 145 nt) and a significant shift on the second peak (around 300 nt). Moreover, we respectively performed Gaussian smoothing to the mutant and wild-type fragments of each SNV and Indel, whose numbers of mutant fragments were larger than 20, and identified their peak lengths where the maximum probability densities were achieved (Fig. 4b). 83.9% of the mutations showed smaller peak lengths of mutant fragments than those of wild-type fragments. Based on the above Gaussian smoothing, we calculated a theoretical curve of proportions of the mutant fragments over wild-type fragments with different sizes. We hypothesized that mutant fragments could be enriched in small fragments smaller than 145 nt.

To validate this hypothesis, we selected four ctDNA samples with L858R mutation and measured their AFs in small fragments by ddPCR. The results of ddPCR confirmed that the AFs in the small fragments were higher than those in the total fragments (Fig. 4c). The results were consistent with our theoretical conclusion that some mutations could be enriched in small fragments, and this phenomenon might further extend the detection limit.

## Discussions

Blood ctDNA has been discovered for several years, and the detection of variations on ctDNA using the next-generation sequencing technology has been significantly improved. Though the sensitivity of different methods on various cancers was frequently reported, NGS sequencing results between ctDNAs and tumor tissues has not been well-documented, and the comparison could be noteworthy to understand the advantages and the limits of capturing-based sequencing to better conduct precision medicine.

UC-Seq significantly improves the sensitivity of ctDNA detection on SNVs and Indels. Besides, this method extends the detection limit of AFs down to 0.1% with controllable false positive rates in ctDNA. However, the AFs of tumor-matched mutations in ctDNAs are affected by many variables and are poorly correlated with their AFs in tumor tissues. Since tumors are usually heterogenic, mutations with low AFs may come from minor sub-clones, and these mutations might thus drop below the detection limit in ctDNA. Usually the cutoff of AFs for actionable mutations in tumor tissues is practically set between 5% to 10% [21]. In addition, the patients, whose tumor sequencing showed the L858R mutation with AFs over 9%, were more sensitive to EGFR-TKIs than those whose AFs were below [22]. Our data suggest that the mutations, with AFs in IIIB and IV lung cancer tissues below 5%, are difficult to be detected in ctDNAs, while mutations of AF over 5% can be identified with high sensitivity (92.9%). Hence the sensitivity of UC-Seq at a depth of 10,000× is sufficient to detect the actionable mutations for late-stage lung cancer patients.

However, UC-Seq might not significantly improve the sensitivity in detecting fusions and copy number variations. The detection of fusions relies on finding the split reads and the pairs of reads spanned over the break points, whose proportion is much lower than the real proportion of fusion fragments in the blood because ctDNA fragments are short in blood. Moreover, some fusion break points could also locate in the introns, which might contain a significant number of repeats like SINEs and are hard to design high-specific probes to efficiently capture fusion fragments. Since the tumor content is much lower in ctDNA than that in tissue, it is much more difficult to detect fusions in ctDNA than that in tumor tissues by the same panel.

The detection of copy number variations is determined by the ratios of gene copy numbers in tumor and the ctDNA proportions in cfDNA. A proper mathematical model is also essential to separate the real signals from background noises. Since the ctDNA proportion in cfDNA is usually around 1% from our data, the change from copy number variation is hard to be distinguished from the fluctuation introduced by experiments. Only copy gain with ratios larger than 3.5 can be detected at an acceptable sensitivity (83.3%) in ctDNA.

Tumor mutational burden (TMB) is proved to be correlated with the response of immunotherapies [23]. TMB detection thus becomes clinical needs. TMB is defined as the number of non-synonymous somatic mutations in whole exome sequencing. However the high cost of whole exome sequencing at high coverage hinders the application of TMB in ctDNA. To expand the application of immunotherapies to late-stage cancer patients, where tumor tissues might not be available, the utility of calculating TMB in ctDNA in a small panel has to be evaluated. Firstly, the correlation of TMB between a small panel and whole exome sequencing depends on the size of the panel. It is suggested by the simulation data that it is better to calculate TMB from a panel whose size is larger than 1 Mb to ensure a high Pearson correlation to be larger than 0.9 (Fig. 2a). Secondly, a sufficiently low limit of detecting the SNVs and Indels is indispensable. Otherwise the correlation of TMB between tumor tissues and ctDNA might not be satisfactory. The cutoff of mutant AFs in our study was set to 0.3%. Since the tissue samples contained only part of the clones of tumors while ctDNA could theoretically detect the mutations from all clones as long as their DNAs are released to the blood stream. The mutations below 0.3% detected by ctDNA could come from other clones of tumors than from the clones in the tissue samples, and hence the correlation of TMB between tissue and ctDNA was lowered. Under this cutoff, the correlation of TMB between tumor tissues and ctDNA reached 0.8 considering all samples. The results also show that bTMB from ctDNA could more properly reflect TMB of metastatic tumor tissues (Pearson correlation = 0.9) than that of primary tissues (Pearson correlation = 0.8). Besides, it might not be appropriate to measure the TMB of IIIB patient by ctDNA (Pearson correlation = 0.66) in a small panel of 490 KB. In summary, bTMB from ctDNA is possible to reveal the TMB of tumor tissues and would be better to represent TMB of tumor tissues when a panel size increases to 1 MB.

Several clinical features could significantly affect the concordance between tumor tissues and ctDNA. Firstly, it is reported that the average half-life of ctDNAs after surgery of complete resection was 114 min, while after incomplete resection, the situations might be diverse [24]. In our study, the ctDNA samples collected at least 1 day after surgeries shows the same trend. ctDNAs from patients who had received surgeries on primary sites have only 41% concordance, while those who had received surgeries on metastatic sites have higher concordance of 87%. Besides, ctDNAs from patients, who were receiving inefficacious targeted therapies or chemotherapies with progress diseases, also present high concordance of 91.3% with tumor tissues, though the sample size was small. The results indicate that the concordance between ctDNAs and tumor tissues could be influenced by the efficacy of treatments. Secondly, tumors are always heterogenic and evolving to gain new phenotypes [25]. The composition of clones is dynamic. Different clones might compete for space and resource, and finally some sub-clones may metastasize to other locations [26]. During this process, some clones may even regress to be un-detectable. In the extreme cases, some metastatic cancer patients could not identify the primary sites during pre-treatment evaluation [27]. Among the ctDNA samples from the treatment-naive patients, whose tumor biopsies had been taken at least 2 weeks ago without treatments before ctDNA sampling, the samples from IV patients have significantly poorer concordance (54.1%) with tumor tissues, while the IIIB samples have high concordance (100%), though the sample size is small. Compared with IIIB tumors, IV tumors might have highly active clonal evolution, which causes poor concordance between ctDNAs and tumor tissues when the time intervals between the tissue biopsy and the blood biopsy are more than 2 weeks. In both cases of low concordance, the status of tumors may dynamically change. The data also reveal that UC-Seq of ctDNA has potential to monitor the efficacy of therapies and the clonal evolution of late-stage tumors.

Interestingly, late-stage cancer patients have various cfDNA concentrations. Though most late-stage lung cancer patients have high cfDNA concentrations, some patients might still have low cfDNA concentrations (< 9 ng/ml) close to the level of healthy people. The ctDNA samples with low concentrations show much worse concordance than those with high concentrations. Moreover, the mutant AFs in ctDNA samples with high concentrations are generally higher (Fig. 3d). That reveals higher proportions of DNA fragments from tumor cells. The cfDNA concentrations are related to the cancer stages and severity. Clinically analysis showed that cfDNAs concentrations of NSCLC patients are higher than benign lung nodules [28]. Besides, the advanced NSCLC patients with low cfDNA concentrations have better overall survival than those with high cfDNA concentrations [29, 30]. Nevertheless, treatments or exercises could significantly affect cfDNA concentrations. It is reported that cfDNA concentrations are elevated up to 15 folds after strenuous exercises due to acute aseptic inflammation [31, 32]. Conversely, people who have chronic occupational exposure to low-dose gamma-neutron and tritium β radiation present lower cfDNA concentrations, due to elevated levels of DNase and antibodies to DNAs in blood [33]. Hence a standard practice of cfDNA sampling have to be established, from which the cfDNA concentrations can be applied to measure the status of patients and the sensitivity of detection can be assured.

Two studies have reported that the mutant fragments from tumors were generally shorter than those of wild-type fragments [12, 34]. With a larger set of clinical samples, our data concurred with their findings and further extended the knowledge. Indicated by wild-type fragments in both patients and healthy donors, the lengths of cell-free DNA fragments have two peaks: One sharp peak at around 170 nt and one broad peak around 320 nt. The core of nucleosome consists of 146 nt of DNA plus up to 80 nt linker DNA regions [35], which creates the sharp peak around 170 nt. The broad peak at around 320 nt is likely to be the length of DNA protected by a dimer of nucleosomes. It is intriguing that mutant fragments were shifted shorter at the first peak and the second peak. 86% of SNVs and Indels had higher mutant AFs in small fragments (< 145 nt), including important actionable mutations such as L858R and exon 19 deletion in EGFR. However, the shortening effect of mutant fragments from tumors is likely to be gene-specific or even position-specific. Though most mutations showed a shortening trend, a considerable number of mutations have indistinguishable length distribution, or larger fragment sizes compared with wild-type fragments. The length distribution of mutant fragments from tumors might be affected by the DNA accessibility of the loci in the tumor tissues. Loci with high DNA accessibility are usually bound by fewer number of nucleosomes [36], and thus present higher odds to be digested by DNases in blood. Furthermore, nucleosome depletion occurs at active transcribing regions [37]. The shortening extent of mutant fragments from tumors might reflect the transcriptional activity of genes in the tumors, especially in a panel that genes are selected from known drug targets and cancer drivers. This finding offers a plausible way to further increase the sensitivity of detecting mutations in ctDNA, and also a theoretical guide for deducing the expressions of genes in tumors.

## Conclusions

In late-stage lung cancer patients, ctDNA generally presented high concordance with tumor tissues. Considering tumor tissues as golden standard of mutation detection, our UC-seq method achieved overall 93.6% sensitivity for SNVs and Indels, and 0.8 Pearson correlation between tumor TMB and bTMB. Efficacious treatments and long sampling date (more than 2 weeks) between tumor tissues and ctDNA could significantly decrease the concordance between ctDNA and corresponding tumor tissues, revealing that ctDNA could dynamically monitor the status of tumors. Besides, low cfDNA concentration could impair the detection of mutations in ctDNA. Furthermore, about 84% mutations showed shorter mutant fragment length than that of wild-type fragments, and their allelic frequencies increased in small-size ctDNA. This finding shows a possible method to further extend the detection limit. Overall, our findings extend the knowledge on ctDNA and may improve its practices in precision medicine.

## Additional files

Additional file 1: **Figure S1.** UC-Seq significantly improve the sensitivity of mutation detection in ctDNA. (A) Sensitivity of ctDNA detection with or without barcoding. (B) Distribution of mutant allelic frequencies (AFs) in ctDNA with or without barcoding. (PDF 756 kb)

Additional file 2: **Figure S2.** The sensitivity of copy gain detection was affected by the copy gain ratio in corresponding tumor tissues. (A) The curve of sensitivity varied with the decreasing of the copy gain ratio cut-offs in corresponding tumor tissues. (B) Scatter plot of maximum MAFs in ctDNA versus copy gain ratio in corresponding tumor tissues. (PDF 868 kb)

## Abbreviations

AF: Allelic frequency
AJCC: American Join Committee
ARMS: Amplification-refractory mutation system
AUC: Area under curve
bTMB: Blood Tumor mutational burden
cfDNA: Cell-free DNA
CNV: Copy number variation
ctDNA: Circulating tumor DNA
ddPCR: Droplet digital PCR
DNA: Deoxyribonucleic acid
Indel: Small insertion and deletion
NGS: Next-generation sequencing
NSCLC: Non-small cell lung cancer
PCR: Polymerase chain reaction
ROC: Receiver operating characteristic
SINE: Short interspersed nuclear element
SNV: Single nucleotide variation
TCGA: The cancer genome atlas
TMB: Tumor mutational burden
UC-Seq: Unique identification indexed capturing-based sequencing
UID: Unique identifier
WES: Whole exome sequencing
WGS: Whole genome sequencing
