# Supplementary material for: The characteristics of ctDNA reveal the high complexity in matching the corresponding tumor tissues
Source: BMC Cancer. 2018 Mar 23;18:319. doi: 10.1186/s12885-018-4199-7 (PMC5865353; doi:10.1186/s12885-018-4199-7)
Supplement: Supplementary file 1 — Figure S1. UC-Seq significantly improve the sensitivity of mutation detection in ctDNA. (A) Sensitivity of ctDNA detection with or without barcoding. (B) Distribution of mutant allelic frequencies (AFs) in ctDNA with or without barcoding. (PDF 756 kb) [file 12885_2018_4199_MOESM1_ESM.pdf]

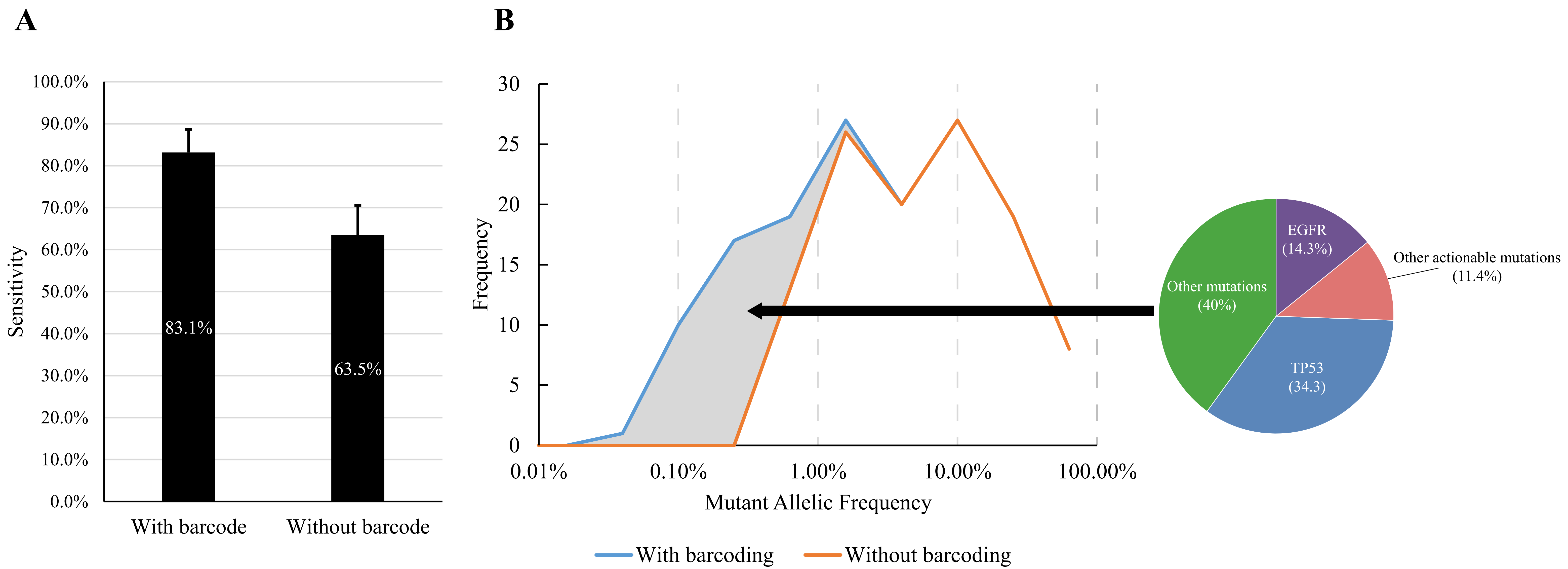

Figure S1. UC-Seq significantly improve the sensitivity of mutation detection in ctDNA. (A) Sensitivity of ctDNA detection with or without barcoding. (B) Distribution of mutant allelic frequencies (AFs) in ctDNA with or without barcoding.
