# Supplementary figures and images for: The characteristics of ctDNA reveal the high complexity in matching the corresponding tumor tissues
Source: BMC Cancer. 2018 Mar 23;18:319. doi: 10.1186/s12885-018-4199-7 (PMC5865353; doi:10.1186/s12885-018-4199-7)

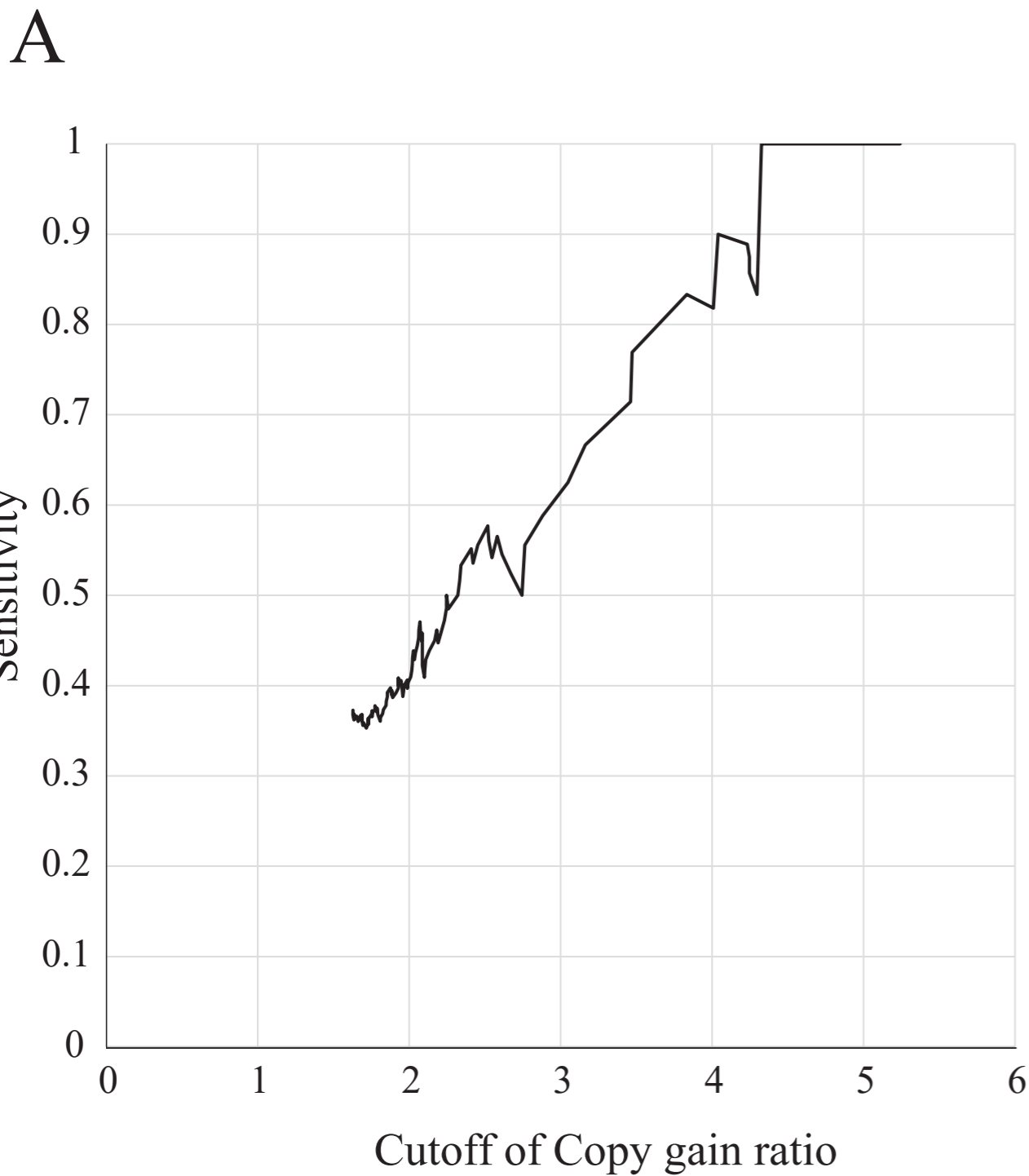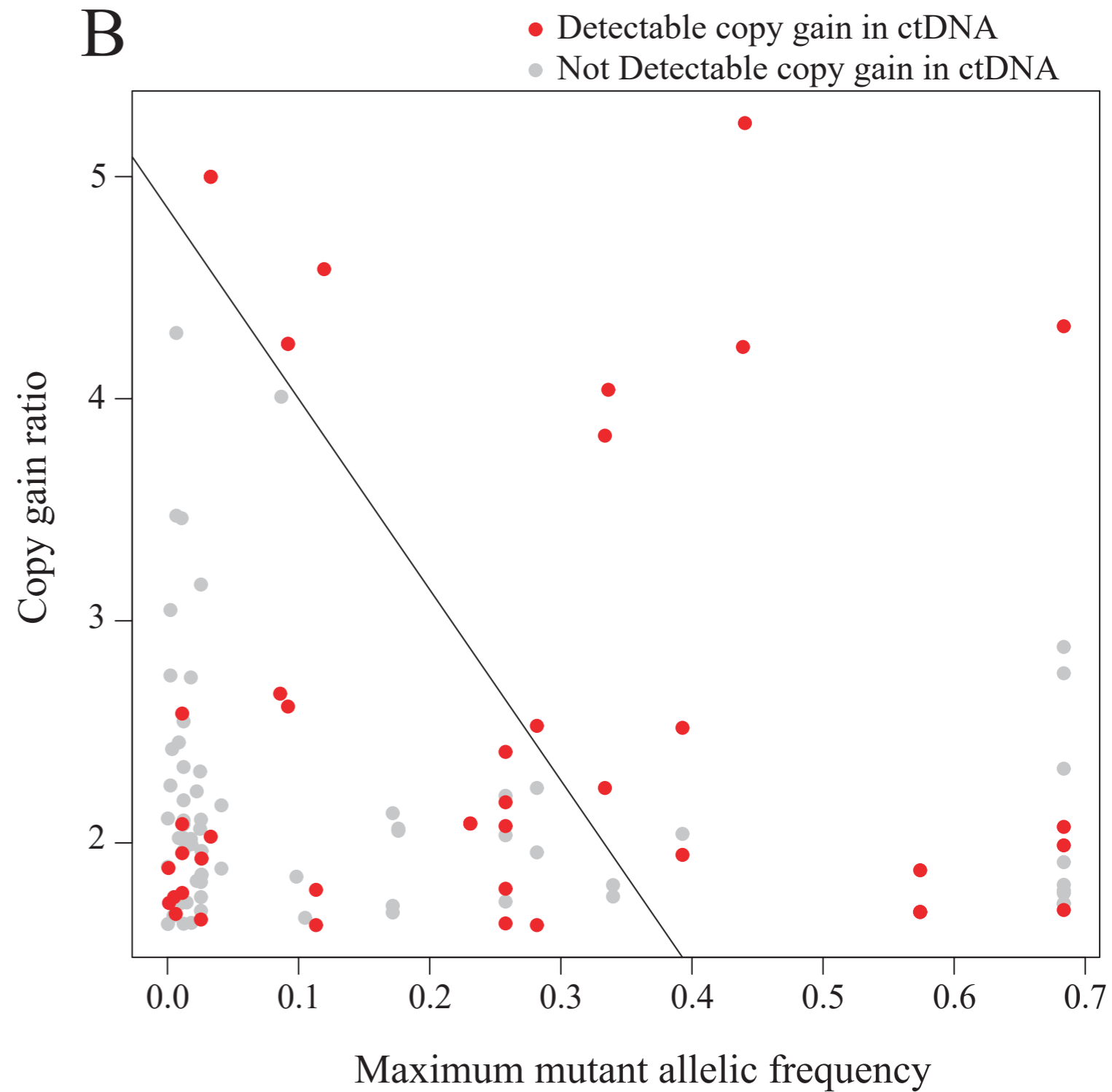

Supplement: Supplementary file 2 — Figure S2. The sensitivity of copy gain detection was affected by the copy gain ratio in corresponding tumor tissues. (A) The curve of sensitivity varied with the decreasing of the copy gain ratio cut-offs in corresponding tumor tissues. (B) Scatter plot of maximum MAFs in ctDNA versus copy gain ratio in corresponding tumor tissues. (PDF 868 kb) [file 12885_2018_4199_MOESM2_ESM.pdf]
